# Volatile and Amino Acid Profiling of Dry Cured Hams from Different Swine Breeds and Processing Methods

**DOI:** 10.3390/molecules18043927

**Published:** 2013-04-03

**Authors:** Diego L. García-González, Ramón Aparicio, Ramón Aparicio-Ruiz

**Affiliations:** Instituto de la Grasa (CSIC), Padre García Tejero, 4, Sevilla E-41012, Spain; E-Mails: aparicio@cica.es (R.A.); aparicioruiz@cica.es (R.A.-R.)

**Keywords:** dry-cured ham, volatile compounds, amino acids, aroma, HS-GCMS, HPLC

## Abstract

The flavor of dry cured ham explains the high appreciation of this product and it determines consumer acceptance. Volatile compounds provide valuable information about the odor and sensory quality of dry cured hams. Since amino acids are the origin of some volatile compounds of dry cured ham, the volatile and amino acid compositions of forty-one dry cured hams from Spain and France were determined to establish associations between them. The samples included different pig breeds (non Iberian *vs.* Iberian), which were additionally affected by different maturation times and feeding types (acorn *vs.* fodder). Results showed that 20 volatile compounds were able to distinguish Iberian and non Iberian hams, and 16 of those had relevant sensory impact according to their odor activity values. 3-Methylbutanol, 2-heptanol and hexanal were among the most concentrated volatile compounds. In the case of non-volatile compounds, the concentrations of amino acids were generally higher in Iberian hams, and all the amino acids were able to distinguish Iberian from non Iberian hams with the exception of tryptophan and asparagine. A strong correlation of some amino acids with volatile compounds was found in the particular case of alcohols and aldehydes when only Iberian hams were considered. The high correlation values found in some cases proved that proteolysis plays an important role in aroma generation.

## 1. Introduction

The unique flavor of dry cured ham is the result of a long manufacturing process that produces changes in its aroma and taste. The dry cured ham aroma is markedly affected by the raw material and several parameters during the production process. Raw hams undergo several stages, such as salting with dry salt, washing, post-salting for salt equalization and ripening-drying. In the course of this long process, over 24 months in some cases, the temperature and humidity are controlled to reduce the risk of bacterial spoilage [1]. However, the final products obtained by this process are heterogeneous and there are many kinds of hams whose overall quality depends on diverse factors such as pig breed, age and feeding. The heterogeneity also occurs within a single ham sample since locations in the ham (*i.e.*, muscles and fat) are characterized by diverse concentrations of flavor compounds. 

The aroma of dry-cured hams is due to the presence of many volatile compounds, most of them produced by chemical and enzymatic mechanisms during the *post-mortem* process [2]. Lipolysis and proteolysis are the main biochemical reactions involved in the generation of these compounds, producing a wide range of volatiles and precursors [3]. Therefore, a better understanding of dry-cured ham aroma should include the identification and quantification of the volatile compounds present in the samples. Several studies have reported information on the volatile composition of Iberian [4], Parma [5], French [6] and Spanish Serrano hams [2]. Some of the published papers have pointed out the variability of the quantified volatiles, which can be due to the fact that the hams are not homogeneous products and the muscles and subcutaneous fat of the samples may differ in their composition. Furthermore, it has been established that chemical changes occurring in different muscles during ripening influence the ham flavor [7]. Nevertheless, the contribution of the main volatiles of each muscle to the ham aroma is scarcely unknown [8]. 

The study of the aroma, and the volatile compounds of dry cured hams, implies the examination of the factors that leads to characteristic sensory notes. Pig feeding and breeding are among the most remarkable factors affecting dry cured ham aroma [9]. Large farm pigs are usually fed with fodder (e.g., mixture of maize with other cereals), while higher quality is achieved if they are fed with acorns instead. Pig breed is also an outstanding characteristic that determine price and quality. Usually the pigs that are fed with acorns are also from the particular Iberian breed. Therefore, the feeding and breed types are two factors that are usually studied together (Iberian+acorn feeding *vs.* non Iberian+fodder feeding).

This work displays the results of a thorough study of the volatiles produced by each one of the four well-known ham locations (*biceps femoris, semimembranosus* and *semitendinosus* muscles, and subcutaneous fat) in hams corresponding to different feeding and breed types. Amino acids, on the other hand, are not only responsible for texture, and partially for taste, of hams, but also they are at the origin of some volatile compounds determined in the muscles [10,11]. For that reason, amino acids, together with creatine and creatinine, have been determined in dry cured hams to find their relationship with the occurrence of some volatile compounds. Although some previous studies have addressed the simultaneous analyses of volatiles and amino acids of dry cured hams, they are based on few samples and/or centered in a particular breed/feeding type [12,13]. The objectives of this work were: (i) the evaluation of the amount of each volatile produced at each ham location, (ii) the understanding of the potential contribution of the volatiles released in these locations to dry cured-ham aroma, and (iii) the relationship between the concentration of amino acids and volatiles quantified in the 4 ham locations. The work was carried out with Spanish (Iberian and non Iberian breeds) and French (non Iberian breeds) hams. The characterization of the samples was carried out with the assistance of statistical procedures taking into account only a classification criterion: Iberian *vs.* non Iberian hams. Information from odor threshold and GC-sniffing/olfactometry (henceforth, GC-O) was taken into consideration for the chemical interpretation of dry cured ham aroma.

## 2. Results and Discussion

The high heterogeneity of ham samples is undoubtedly the main hurdle towards a representative aroma analysis. In order to obtain a representative sample from the ham pieces, 350 g of the part located along and behind the femur was collected from each one of the above described French and Spanish hams. The samples were collected from four well differentiated locations: *biceps femoris* (BF), *semimembranosus* (SM) and *semitendinosus* (ST) muscles, and subcutaneous fat (SF). 

### 2.1. Amino Acids and Related Compounds

Dry-cured hams contain a large number of free amino acids and derivatives resulting from extensive proteolysis, which is characteristic of all types of hams, though the extent of amino acid release depends on the processing time [14,15]. The responsible enzymes are aminopeptidases that act on the N-terminal of peptides and proteins [14]. Table 1 shows that lysine, followed by others such as valine, isoleucine, leucine and phenylalanine, are the amino acids determined in higher amounts. The values of amino acids are, in general, higher in Iberian hams, which explains that all the amino acids can be used to distinguish Iberian from non Iberian hams with the exception of tryptophan and asparagine. Thus, results show leucine, isoleucine and lysine are higher in Iberian hams whose drying step is longer, which agrees with Toldrá *et al.* [15].

Toldrá [3] described that glutamic acid, aspartic acid, histidine, arginine, valine, methionine, isoleucine, leucine, tryptophan and lysine were strongly correlated with the length of the drying process [3]. Our results agree with those results, except for tryptophan, when hams were classified into four groups: 7 months, 8–11 months, 12–17 months and more the 18 months (Iberian hams). Those amino acids were positively correlated (*p* < 0.001) to the drying length with adjusted-R^2^ regression coefficients higher than 0.70. Coefficient values between 0.65 and 0.70 were also determined for tyrosine, aspargine, taurine, serine, and glycine.

### 2.2. Volatile Compounds

**Figure 1:**
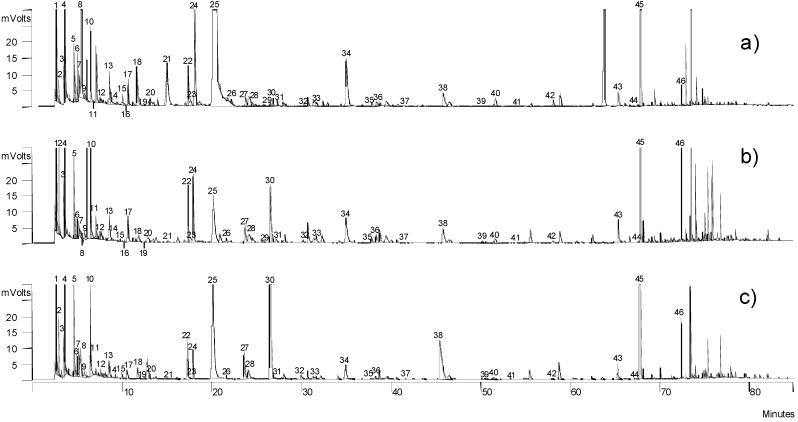
Chromatograms of volatiles of the subcutaneous fat of two non Iberian hams from different geographical origins: (**a**) Iberian ham from PDO “Jamón de Huelva”; (**b**) Non Iberian ham from PDO “Jamón de Teruel”; and (**c**) Non Iberian ham from Parlan, Auvergne (France). Note: codes are described in Table 1. Chromatographic method is described in Experimental section.

Table 2 shows the volatile compounds identified in the samples clustered according to their breeding (non Iberian and Iberian), while Figure 1 displays the chromatograms of three dry-cured hams. The formation of these volatiles is caused by the intense degradation processes that happen in lipids and proteins during the processing of dry-cured hams. Most of the volatiles identified (hydrocarbons, aldehydes, alcohols, ketones, esters, carboxylic acids, *etc.*) derive from lipid oxidation that is probably the main source of ham volatiles as in almost all fat products [16]. Other volatiles, such as 3-methylbutanol and benzaldehyde, derive from reactions between amino acids and reducing sugars referred to as the Maillard reaction [16]. An intense proteolysis, and hence a larger release of amino acids in hams, promotes the Strecker degradation, and is the implication of reactive carbonyls in the Strecker degradation of free amino acids, which would explain the concentrations of the branched aldehydes (e.g., 2-methylbutanal derived from isoleucine) quantified in dry-cured hams.

**Table 1:** Amino acids and related compounds (mg/100g) determined in non Iberian and Iberian hams. Column with *p*-value lower than 0.05 indicates amino acids distinguish between hams. Values are expressed as the mean and the standard error of the mean.

Code	Compound	Non Iberian ^a^	Iberian ^a^	*p*
A1	Tryptophan	23 ± 1	21 ± 1	0.30
A2	Phenyalanine	252 ± 55	286 ± 67	<0.01
A3	Tyrosine	103 ± 3	150 ± 11	<0.01
A4	Tyramine	12 ± 2	2 ± 1	0.02
A5	Isoleucine	300 ± 8	391 ± 10	<0.01
A6	Leucine	267 ± 7	353 ± 11	<0.01
A7	Methionine	157 ± 1	209 ± 1	<0.01
A8	Valine	316 ± 1	402 ± 1	<0.01
A9	Creatine	1637 ± 19	118 ± 23	<0.01
A10	Proline	146 ± 4	197 ± 6	<0.01
A11	Creatinine	29 ± 1	36 ± 1	<0.01
A12	Glutamic acid	543 ± 162	739 ± 25	<0.01
A13	Arginine	195 ± 6	301 ± 9	<0.01
A14	Asparagine	185 ± 50	195 ± 15	0.50
A15	Taurine	57 ± 1	86 ± 8	<0.01
A16	Histidine	165 ± 5	226 ± 6	<0.01
A17	Serine	181 ± 7	255 ± 5	<0.01
A18	Glycine	92 ± 3	119 ± 2	<0.01
A19	Lysine	686 ± 22	966 ± 29	<0.01

Note: ^a^ mean ± standard deviation.

**Table 2:** Codes and relative retention times (Rt) of the volatile compounds quantified in the hams, mean concentration and standard deviation of volatiles determined in non Iberian and Iberian hams, and *p* values of each volatile compound classifying the hams by their breeds (non Iberian *vs.* Iberian). Odor threshold values (OT) in mg/kg and sensory descriptions obtained by GC-olfactometry (GC-O) are displayed as well.

Code	Rt	Volatile compound	Non Iberian ^a^	Iberian ^a^	*p*	OT	GC-O
V1	0.16	Hexane	0.36 ± 0.03	0.29 ± 0.03	0.20	1.50	Spicy
V2	0.17	Heptane	0.22 ± 0.03	0.23 ± 0.03	0.85	0.67	Sweety, alkane
V3	0.20	Octane	2.23 ± 0.42	3.00 ± 0.41	0.35	0.94	Sweety, alkane
V4	0.21	2-Propanone	1.72 ± 0.13	2.21 ± 0.32	0.10	500	Fruity, apple, cooked meat
V5	0.27	2-Butanone	0.34 ± 0.04	0.20 ± 0.02	0.03	40	Ethereal
V6	0.29	3-Methylbutanal	0.13 ± 0.02	0.38 ± 0.06	<0.01	0.08	Acorn, fruity, cheesy, salty
V7	0.31	2-Propanol	0.07 ± 0.01	0.07 ± 0.01	0.95	26	Alcoholic, dry, buttery-taste
V8	0.32	Ethanol	1.31 ± 0.20	1.52 ± 0.26	0.61	30	Alcohol, sweet
V9	0.34	2-Ethylfuran	0.09 ± 0.04	0.07 ± 0.01	0.74	-	Sweet
V10	0.38	2-Pentanone + 3-Pentanone	0.79 ± 0.08	0.48 ± 0.10	0.05	70 ^b^	Sweet, fruity, green
V11	0.39	2,3-Butanedione	0.36 ± 0.07	0.35 ± 0.10	0.95	-	Vanilla/caramel-like, buttery
V12	0.46	α-Pinene	0.09 ± 0.01	0.08 ± 0.02	0.57	0.02	Sharp, pine
V13	0.51	Methyl benzene	0.10 ± 0.01	0.12 ± 0.01	0.03	0.33	Plastic, glue, strong
V14	0.53	2-Methyl-3-buten-2-ol	0.06 ± 0.01	0.07 ± 0.01	0.74	0.48	Earthy
V15	0.60	Dimethyl disulfide	0.02 ± 0.00 ^c^	0.02 ± 0.00 ^c^	0.39	0.01	Cauliflowers, vegetable
V16	0.61	Butyl acetate	0.01 ± 0.00 ^c^	0.01 ± 0.00 ^c^	0.80	0.30	Fruity, banana, apple
V17	0.64	Hexanal	1.18 ± 0.20	3.76 ± 0.64	<0.01	0.08	Green, grassy, fatty
V18	0.69	2-Methyl propanol	0.11 ± 0.01	0.32 ± 0.04	<0.01	1.00	Wine, penetrating
V19	0.75	2-Butanol	0.03 ± 0.01	0.02 ± 0.00	0.16	0.50	Winey
V20	0.78	Ethyl benzene	0.03 ± 0.00 ^c^	0.02 ± 0.00 ^c^	0.81	-	Dry, glue, unpleasant
V21	0.90	Butanol	0.04 ± 0.02	0.15 ± 0.01	0.01	0.04	Fruity, medicinal
V22	1.05	2-Heptanone	1.56 ± 0.17	1.24 ± 0.28	0.36	0.30	Spicy, acorn, blue cheese
V23	1.06	Heptanal	1.03 ± 0.33	1.47 ± 0.21	0.49	0.50	Fatty, greasy, ham-like
V24	1.09	Limonene	0.59 ± 0.10	2.68 ± 0.56	<0.01	0.25	Citric, fresh
V25	1.21	3-Methylbutanol	1.33 ± 0.11	5.27 ± 1.15	<0.01	0.10	Woody, acorn, pleasant green
V26	1.31	2-Pentylfuran	0.49 ± 0.08	0.94 ± 0.22	0.01	0.10	Green fruity, butter
V27	1.43	Octan-3-one	1.25 ± 0.07	0.63 ± 0.08	<0.01	0.01	Spicy, mushroom, dirty
V28	1.46	Pentanol	1.25 ± 0.15	1.26 ± 0.06	0.99	0.47	Pungent, strong, balsamic
V29	1.59	(*E*,*E*)-2,4-Decadienal	0.46 ± 0.06	0.03 ± 0.01	<0.01	2.50	Fatty, rancid
V30	1.61	2-Octanone	2.20 ± 0.37	0.72 ± 0.12	0.03	0.51	Fruity, floral, green, fresh
V31	1.63	Octanal	4.28 ± 0.88	7.38 ± 1.10	0.04	0.32	Meat-like, green, fresh
V32	1.84	(*E*)-2-Heptenal	1.33 ± 0.33	0.71 ± 0.19	0.31	0.05	Green, fatty, fruity, almonds
V33	1.89	2-Heptanol	0.55 ± 0.06	0.70 ± 0.13	0.27	0.01	Oily, sweety
V34	2.09	Hexanol	1.74 ± 0.18	4.10 ± 0.59	<0.01	0.40	Fruity, green
V35	2.30	2-Nonanone	1.13 ± 0.22	1.69 ± 0.52	0.26	0.10	Floral, fruity, blue cheese
V36	2.33	Nonanal	2.50 ± 0.44	4.33 ± 0.67	0.04	0.15	Rancid, fatty, tallowy
V37	2.55	(*E*)-2-Octenal	0.84 ± 0.16	2.47 ± 0.58	<0.01	0.00 ^d^	Leaves, pungent, fatty, fruity
V38	2.76	1-Octen-3-ol	2.72 ± 0.20	1.66 ± 0.21	0.01	0.00 ^d^	Mushroom-like, earthy, dust
V39	3.02	Decanal	0.26 ± 0.02	0.28 ± 0.03	0.74	0.65	Citrus, waxy
V40	3.11	Benzaldehyde	0.97 ± 0.09	1.78 ± 0.21	<0.01	0.06	Bitter almonds, penetrating
V41	3.22	(*E*)-2-Nonenal	2.03 ± 0.37	3.88 ± 0.92	0.03	0.15	Fatty, waxy
V42	3.47	Octanol	0.42 ± 0.05	0.98 ± 0.12	<0.01	0.03	Fatty, sharp
V43	3.94	Butanoic acid	0.57 ± 0.05	0.61 ± 0.09	0.69	0.65	Cheesy, rancid
V44	4.13	Nonanol	0.19 ± 0.01	0.26 ± 0.04	0.08	0.28	Fatty green
V45	4.14	Isobutyric acid	6.49 ± 0.46	4.99 ± 0.67	0.12	8.10	Iron, fishy
V46	4.35	Hexanoic acid	4.76 ± 0.58	4.90 ± 0.88	0.12	0.70	Fatty, cheese, sweaty

Note: ^a^ mean ± standard deviation; ^b^ odor threshold of 3-pentanone; ^c^ standard deviations dimethyl disulfide (0.004 and 0.003), butyl acetate (0.001 and 0.001), 2-butanol (0.002), and ethyl benzene (0.003 and 0.003); ^d^ E-2-Octenal (0.004), and 1-octen-3-ol (0.001).

In terms of total concentrations of the most abundant series (alcohols, aldehydes, hydrocarbons and ketones) quantified in Iberian hams, the highest concentrations corresponded to alcohols followed by ketones, aldehydes and hydrocarbons [17], while ketones showed the highest concentrations in non Iberian hams, followed by alcohols, aldehydes and hydrocarbons [18]. The concentration of these compounds depends on the location where they are quantified. Thus, Table 3 shows the concentration of the volatile compounds quantified in the four ham locations displayed in chemical series.

**Table 3:** Volatile compounds (mg/kg) quantified in four locations of dry cured ham samples (SF, subcutaneous fat, BF, *biceps femoris*, SM, *semimembranosus*, ST *semitendinosus* muscles) and distributed in chemical series. Presented data are mean values from all samples (non Iberian and Iberian), while figures within brackets are minimum and maximum concentrations.

Chemical series	Volatile compounds	BF ^a^	SF ^a^	SM ^a^	ST ^a^
Hydrocarbons	Hexane	0.40 (0.03–1.77)	0.27 (0.04–0.66)	0.37 (0.03–0.97)	0.38 (0.04–1.25)
	Heptane	0.16 (0.03–1.13)	0.26 (0.05–0.70)	0.29 (0.03–2.99)	0.15 (0.02–0.58)
	Octane	1.66 (0.10–7.91)	3.22 (0.52–10.81)	2.12 (0.39–5.30)	1.37 (0.40–3.81)
	Methyl benzene	0.14 (0.04–0.37)	0.15 (0.02–0.44)	0.19 (0.07–0.42)	0.13 (0.06–0.24)
	Ethyl benzene	0.12 (tr-0.35)	0.20 (tr-0.73)	0.17 (0.01–0.74)	0.14 (0.01–0.71)
	Limonene	0.40 (0.01–4.71)	0.51 (tr-3.16)	1.42 (tr-14.60)	0.56 (tr-5.14)
	α-Pinene	0.19 (tr-1.61)	0.10 (tr-0.82)	0.28 (tr-1.38)	0.22 (0.02–1.56)
Alcohols	2-Propanol	0.32 (0.01–2.64)	0.11 (tr-0.52)	0.33 (tr-3.25)	0.29 (tr-1.62)
	Ethanol	0.70 (0.01–5.86)	0.40 (0.01–3.52)	0.83 (0.01–8.90)	0.79 (0.01–5.46)
	2-Methyl-3-buten-2-ol	0.04 (tr-0.24)	0.11 (tr-0.42)	0.04 (tr-0.19)	0.05 (tr-0.40)
	2-Methyl propanol	0.13 (tr-0.63)	0.08 (tr-0.26)	0.15 (0.01–0.75)	0.12 (tr-0.70)
	2-Butanol	0.02 (tr-0.27)	0.03 (tr-0.13)	0.03 (tr-0.49)	0.03 (tr-0.40)
	Butanol	0.28 (tr-8.50)	0.05 (tr-0.16)	0.09 (tr-0.37)	0.09 (tr-0.79)
	3-Methyl butanol	3.10 (0.06–21.31)	0.65 (0.04–6.13)	1.76 (0.02–1 0.63)	2.04 (0.02–17.99)
	Pentanol	1.19 (0.07–7.66)	1.48 (0.28–4.81)	1.26 (0.09–7.43)	1.17 (0.11–8.57)
	2-Heptanol	0.66 (tr-5.06)	0.71(0.04–3.12)	0.72 (0.09–3.76)	0.66 (0.05–3.27)
	Hexanol	1.56 (0.14–11.31)	2.84 (0.03–10.13)	2.10 (0.45–6.32)	2.55 (0.34–9.77)
	1-Octen-3-ol	2.48 (tr-7.73)	2.27 (0.07–8.40)	0.54 (0.11–1.65)	0.65 (0.09–2.54)
	Octanol	0.56 (tr-3.02)	0.79 (0.13–2.90)	0.52 (tr-2.13)	0.53 (tr-2.25)
	Nonanol	0.23 (0.02–1.02)	0.38 (0.08–0.92)	0.19 (0.03–0.49)	0.19 (0.02–1.09)
Aldehydes	3-Methylbutanal	0.20 (0.02–1.23)	0.13 (0.01–046)	0.40 (tr-2.23)	0.21 (0.01–1.19)
	Hexanal	0.80 (0.05–.41)	3.29 (0.03–15.58)	1.20 (0.08–11.40)	0.74 (0.06–7.17)
	Heptanal	0.82 (tr-6.11)	2.51 (0.01–8.76)	0.87 (tr-4.01)	0.77 (tr-4.89)
	(*E,E*)-2,4-Decadienal	0.36 (tr-2.45)	0.25 (tr-1.56)	0.26 (tr-2.97)	0.43 (tr-4.80)
	Octanal	1.70 (tr-8.60)	1 0.45 (0.04–37.40)	5.10 (0.04–28.46)	4.11 (tr-30.95)
	(*E*)-2-Heptenal	1.89 (0.01–50.59)	1.68 (0.01–15.22)	2.69 (0.04–10.67)	1.97 (0.01–35.65)
	Nonanal	4.57 (tr-67.17)	5.05 (0.03–18.97)	4.91 (tr-23.51)	4.05 (tr-31.84)
	(*E*)-2-Octenal	0.43 (0.02–3.01)	2.23(0.03–18.61)	0.59 (0.02–7.65)	0.33 (0.02–3.24)
	Decanal	0.17 (tr-0.82)	0.18 (tr-0.77)	0.20 (0.05–0.57)	0.16 (tr-2.15)
	Benzaldehyde	1.27 (0.21–5.74)	0.86 (0.12–2.29)	1.54 (0.01–5.42)	1.10 (0.2–3.84)
	(*E*)-2-Nonenal	1.14 (0.01–9.22)	4.64 (0.14–25.14)	1.18 (0.14–8.18)	0.87 (0.08–7.94)
Ketones	2-Octanone	1.89 (tr-13.36)	0.93 (tr-4.17)	0.67 (tr-7.27)	1.88 (tr-16.27)
	2-Propanone	2.88 (0.09–9.45)	1.53 (0.12–6.67)	2.73 (0.301 0.29)	2.78 (0.03–12.47)
	2-Butanone	0.28 (0.02–1.16)	0.13 (0.05–0.30)	0.41 (0.01–2.34)	0.27 (0.08–0.82)
	2-Pentanone ^b^	0.70 (tr-2.46)	0.57 (tr-3.54)	0.70 (tr-3.67)	0.80 (tr-3.82)
	2,3-Butanedione	0.47 (tr-7.51)	0.57 (tr-3.83)	0.41 (tr-6.84)	0.47 (tr-4.71)
	2-Heptanone	185 (tr-7.77)	1.53 (tr-10.14)	2.01 (0.20–9.30)	2.45 (tr-14.48)
	Octen-3-one	0.77 (0.05–2.25)	0.89 (0.08–2.44)	1.07 (0.02–3.19)	0.79 (tr-2.01)
	2-Nonanone	1.46 (0.08–1 0.73)	2.47 (tr-11.16)	2.17 (0.15–18.46)	2.31 (0.20–13.75)
Acids	Butanoic acid	0.47 (0.03–1.48)	0.64 (0.04–2.67)	0.49 (0.06–1.78)	0.56 (0.06–1.72)
	Isobutyric acid	6.10 (0.48–20.72)	4.45 (0.32–11.55)	4.41 (0.50–17.58)	5.37 (0.56–12.85)
	Hexanoic acid	2.70 (0.24–18.07)	7.60 (0.10–28.98)	4.01 (0.25–24.37)	4.39 (0.27–44.19)
Furans	2-Pentylfuran	0.34 (tr-2.28)	1.15 (0.03–5.09)	0.63 (tr-3.09)	0.23 (tr-1.43)
	2-Ethylfuran	0.17 (tr-5.58)	0.11 (0.01–0.29)	0.09 (tr-0.31)	0.06 (tr-0.28)
Esters	Butyl acetate	0.03 (tr-0.28)	0.01 (tr-0.04)	0.02 (tr-0.07)	0.02 (tr-0.07)
Sulfur compounds	Dimethyl disulfide	0.03 (tr-0.22)	0.02 (tr-0.13)	0.05 (tr-1.10)	0.03 (tr-0.14)

Note: ^a^ mean (minimum-maximum) values; ^b^ 3-pentanone; tr, traces.

#### 2.2.1. Hydrocarbons

The concentration of the volatile compounds distributed in the different chemical series pointed out that the amount of hydrocarbons was higher in SM and SF and lower in BF and ST. Among the hydrocarbons, limonene and octane were the most abundant in the four locations. Hydrocarbons were at higher concentrations in SF than in the muscles with the exception of limonene and α-pinene. According to diverse authors [19,20], the presence of limonene in the hams has been associated with the pig feeding and it contributes with “lemon” sensory notes to ham flavor. Taking into account the odor thresholds, octane and limonene are, among the quantified hydrocarbons, the major contributors to ham odors although they do not contribute in same manner to all the hams. Thus, while the octane concentrations are higher than its odor threshold (OT) in samples from all breeds—Table 2 shows high *p*-value when comparing non Iberian *vs.* Iberian hams, the concentration of limonene has been quantified in amounts higher than its OT in all the Iberian hams, whichever the analyzed muscle, the highest values corresponding to SM (Table 3). Limonene, in concentrations higher than its OT, were also determined in all the muscles of Gascon and Basque hams, which agrees with Sabio *et al.* [19], and in two samples from PDO Teruel. This compound always contributed to lemon odor in hams with a maturation time higher than 12 months (Iberian hams maturation time was ≥ 18 months). Limonene was not perceived, on the contrary, with hams with short processing times, such as some American hams [21]. The concentration of α-pinene is higher than its OT in most of the samples, which means this compound contributes to ham aroma with the pine sensory note regardless the pig breed. The high concentration of hydrocarbons in Iberian hams was also observed for the total sum of the concentrations of hydrocarbons.

#### 2.2.2. Alcohols

The identified alcohols, linear and branched, were the most abundant volatiles because they are among the main lipid oxidation products. The methyl branched alcohols can also be derived from the Strecker degradation of amino acids. It is known that branched alcohols originate from microbial degradation of the respective branched aldehydes [22]. Thus, the formation and release of branched alcohols is affected by the salting conditions due to the antimicrobial activity of NaCl. Thus, a higher production of branched alcohols is observed when NaCl is partially replaced by other formulations [23].

The highest concentration of total alcohols was found in BF muscle (11.27 mg/kg) while the lowest concentration corresponded to SM (8.56 mg/kg). 3-Methyl-1-butanol was by far the most abundant alcohol in the three muscles. The high concentration of 3-methyl-1-butanol (*i.e*., 3.10 mg/kg in BF) can be due to the activity of the microorganisms present in the ham. Microorganisms can act on 3-methylbutanal formed by Strecker degradation of amino acids during proteolysis to give rise to 3-methylbutanol [24]. The alcohol with the highest concentration in SF was hexanol. In SF, where lipids mean 89.7% [25], most of alcohols are produced by lipid oxidation and the proteolysis mechanism does not occur at great extent. 

Because of the high OT of some alcohols (2-propanol, ethanol, 2-methyl-3-buten-2-ol, 2-mehyl propanol, 2-butanol, and nonanol) in comparison with their concentrations (Table 2), their impact on aroma has been considered minor [26]. Table 2 also shows that, in general, alcohols contribute to ham flavor with herbaceous, woody and fatty notes [27]. 3-Methyl-1-butanol has a significant sensory impact and it is a marker of Iberian hams because its high concentration in these hams compared to other breeds. The contribution of this compound to aroma depends on its concentration as it varies from green [8] to dried fruits, and particularly to acorns in the case Iberian hams (Table 2). Another odor compound whose concentration is higher in Iberian hams is hexanol, which contributes to fruity-green odor perception. Its concentration in the SF is mostly responsible of its odor perception. Among the alcohols which contribute to aroma, pentanol and 2-heptanol, for instance, would not be able to distinguish between non Iberian and Iberian hams (*p* < 0.05). The low odor threshold of 1-octen-3-ol indicates that it contributes with a strong mushroom aroma (Table 2) to almost all the hams. The concentration of this compound significantly rises as the amount of curing salt increases [28], which may explain the differences between samples.

The study of the relationship between alcohols and amino acids showed that there was much more relationship between them in non Iberian than in Iberian hams. The values of the adjusted-R^2^ when regressing both data sets were lower in the former, with the exception of 2-butanol from ST with histidine (0.96). On the contrary, high correlation values between the concentrations of amino acids and volatiles were observed in Iberian hams because the increase of free amino acids in long-processed hams [11]. Thus, we found that 2-methyl propanol was the only alcohol that was not related with amino acids while butanol was highly correlated with tyrosine (0.89) and taurine (0.93). Table 4 shows the correlation values between alcohols and amino acids. Ethanol showed a fine correlation exclusively with creatine for all the analyzed locations while 3-methyl-1-butanol was highly correlated with tyramine (0.92). Creatine was also well correlated with 2-heptanol (0.92) and 1-octen-3-ol (0.93).

**Table 4:** Correlation between alcohols and amino acids determined in Iberian hams with an adjusted-R^2^ regression coefficient higher than 0.75 with *p* < 0.05. Notes: Codes of volatile compounds are described in Table 2; BF, *biceps femoris* muscle; ST, *semitendinosus* muscle; SM, *semimembranosus* muscle; SF, subcutaneous fat.

Amino acids	Volatile compounds											
	V7	V8	V14	V19	V21	V25	V28	V34	V33	V38	V42	V44
Tryptophan										SF:0.75	BF:0.79	BF:0.80
Tyrosine					BF:0.85 ST:0.80 SM:0.89			ST:0.76		ST:0.81		
Tyramine						ST:0.92 SF:0.79						
Creatine		BF:0.85 SF:0.79	BF:0.82						SF:0.89 BF:0.92	BF:0.83 ST:0.79 SM:0.93	BF:0.75	BF:0.82 ST:0.87
Asparagine	BF:0.77											
Taurine			ST:0.82	ST:0.87	BF:0.93		BF:0.81 ST:0.80	ST:0.75				
Glycine								BF:0.85				

#### 2.2.3. Aldehydes

Aldehydes play an important role to the overall flavor of all fat products such as olive oil and dry-cured hams because of their low odor thresholds and high concentrations. SF showed the highest concentration of total aldehydes (21.27 mg/kg) while the lowest concentrations were found in BF (13.35 mg/kg) and ST (14.74 mg/kg) muscles (Table 3). This difference in concentration is due to the fact that saturated and monosaturated aldehydes result from lipid oxidation on the contrary to methyl-branched aldehydes (*i.e.*, 3-methylbutanal), which are derived from the Strecker degradation reactions of free amino acids [29]. Thus, the lowest concentrations of 3-methylbutanal was found in SF, where the protein composition is not relevant (Table 3). 

The most abundant saturated aldehyde is octanal (Table 3) in all the locations, followed by nonanal (responsible for rancid attribute) and hexanal. The two latter are characteristics of rancid lipid matrices [17]. (*E*)-2-heptenal and (*E*)-2-nonenal were also quantified at high concentrations in the muscles. Hexanal is generated from the oxidative decomposition of linoleic acid [30] and its contribution to aroma depends on its concentration. Thus, hexanal at low concentrations has a pleasant and grassy aroma [31], which turns fatty at medium concentration and extremely rancid and tallowy at high concentrations [32]. At the concentrations determined in the analyzed hams, hexanal contributes to grassy odor and, perhaps, to a fatty perception in the case of some Iberian hams. Contrary to be suggested by other authors [33], hexanal is not related to the development of rancid flavors, and experience shows that its rancid aroma cannot be masked by other volatile compounds [32].

The polyunsaturated aldehyde (*E,E*)-2,4-decadienal does not contribute to ham odor due to its high OT (2.50 mg/kg) (Table 2); with the exception of five hams, which presented concentrations higher than OT. Decanal and E-2-nonenal also showed concentrations (Table 3) lower than their OTs (Table 2). The contribution of heptanal and nonanal to aroma is also clear because of their high concentration values in most of the samples compared to their OT. Nonanal compound comes from the oxidation of oleic acid that is the most abundant unsaturated fatty acid in hams [21].

Saturated aldehydes are of utmost importance for the overall aroma of the hams. Lipid oxidation seems to be partially affected by the pig feeding/breed because the concentrations of hexanal, octanal and nonanal are higher in Iberian dry-cured hams than in the other samples (Table 2). In general, saturated aldehydes contribute to aroma with sensory descriptors such as green/grassy (hexanal), ham-like/fatty (heptanal), meat-like/fruity (octanal) and tallowy/rancid (nonanal) although with different intensity depending on the breed (Iberian *vs.* non Iberian hams).

The monounsaturated aldehydes [(*E*)-2-heptenal, (*E*)-2-octenal, (*E*)-2-nonenal] results from lipid oxidation as well. Their concentrations were, in general, lower than those of saturated aldehydes but they contribute to aroma since their concentrations are higher than their OT in many of the samples. Concerning (*E*)-2-heptenal, the number of hams whose concentrations are higher than the corresponding OT is a little lower in SF than in the muscles. Its low OT (0.05 mg/kg) allows contributing to aroma with almond-like, green fruity and fatty sensory notes. The concentration of this compound has not allowed distinguishing non Iberian from Iberian hams, which could mean that these sensory notes would be of similar level in all the hams. The concentrations of (*E*)-2-octenal and (*E*)-2-nonenal are higher in SF but their low OTs (0.004 mg/kg and 0.15 mg/kg respectively) indicate that they contributes to the aroma in all the analyzed locations. Their concentrations allowed distinguishing Iberian from non Iberian hams, and they contribute to the pungent and fatty sensory descriptors of Iberian hams.

In addition to the branched aldehydes, the reaction between amino acids also produces aromatic aldehydes such as benzaldehyde, although the latter can also be formed during lipid oxidation. It contributes substantially to dry-cured ham aroma with a bitter almond sensory note because of its low OT as found by other authors [34] as well. This compound has been found in Iberian hams at a very high concentration (Table 2) up to the point that can be a good marker distinguishing non Iberian from Iberian hams (*p* < 10–4). Authors [35] found a continuous increase in the ratio of benzaldehyde during the ripening time, which can explain its high values in Iberian hams.

Benzaldehyde is another volatile well correlated with different amino acids in ST muscle (e.g., methionine, asparagine, serine). The rest of aldehydes (hexanal, heptanal, (*E*,*E*)-2,4-decadienal, octanal, (*E*)-2-heptenal, nonanal, (*E*)-2-octenal, decanal) are correlated with amino acids with diverse regression coefficients (adjusted-R^2^ ≥ 0.75).

We have applied the mathematical procedure of correlation to determine the possible relationship between amino acids and aldehydes quantified in the four locations of hams. No significant relationships (adjusted-R^2^ > 0.75) were found when the study was centered in the whole set of hams, in which the non Iberian hams mean more than 80%. However studying Iberian hams, whose maturation period is longer, many other relationships between amino acids and volatiles were found. Thus, Table 5 shows that 3-methylbutanal is correlated with diverse amino acids (e.g., methionine and histidine) in the three studied muscles, mainly in ST. However, we have not found a plausible relationship between 3-methyl-butanal and its precursors valine, isoleucine and leucine [15] although the concentrations of the volatile compound and the cited amino acids are much higher in Iberian hams (*p* < 0.01) (Table 1 and Table 2).

**Table 5:** Correlation between aldehydes and amino acids determined in Iberian hams with an adjusted-R^2^ regression coefficient higher than 0.75 with *p* < 0.05. Notes: Codes of volatile compounds are described in Table 2; BF, *biceps femoris* muscle; ST, *semitendinosus* muscle; SM, *semimembranosus* muscle; SF, subcutaneous fat.

Amino acids	Volatile compounds									
	V6	V17	V23	V29	V31	V32	V36	V37	V39	V40
Tryptophan	ST:0.83 SM:0.84									
Phenylalanine		ST:0.88	ST:0.91		ST:0.78			ST:0.77		
Tyrosine			BF:0.81		SF:0.78 BF:0.78					
Methionine	ST:0.89									ST:0.85
Creatine				BF:0.78			BF:0.86			
Creatinine	SM:0.84								BF:0.88	
Arginine		ST:0.87	ST:0.79					ST:0.80		
Asparagine	ST:0.82 BF:0.77									ST:0.79
Taurine		BF:0.94	BF:0.91		BF:0.84	BF:0.82		BF:0.75		ST:0.76
Histidine	ST:0.92									ST:0.75
Serine										ST:0.87
Glycine	ST:0.78									

#### 2.2.4. Ketones

Lipid oxidation, by means of autoxidation or beta-oxidation of fatty acids, is mainly responsible for the production of methyl ketones. These compounds contribute to dry-cured ham aroma [9] and they are considered as responsible for ham fatty aromas associated with cooked meat [36] and the blue cheese sensory note (Table 2). Dirinck *et al.* [37] had already reported that 2-propanone had the highest concentration among the ketones identified in this chemical series, which we have confirmed in the three muscles and subcutaneous fat of the analyzed hams. However, the concentrations of all the methyl ketones, with exception of 2-nonanone, were lower in SF in comparison to the muscles despite they derive from lipid oxidation. In general, the concentration of methyl ketones is higher in Iberian hams though only 2-butanone, 2-pentanone and 2-octanone presented low p-values as to distinguish those from non Iberian hams. Although the concentration of 2-heptanone does not allow distinguishing those hams, this compound contributes to ham aroma (OT ≤ 0.30) with spicy/blue cheese/acorn sensory notes. In fact, the low odor thresholds of these compounds indicate that they are neat contributors to ham aroma with the exception of 2-propanone, 2-butanone. Octen-3-one is a remarkable ketone since its very low OT allows contributing to ham aroma, with floral/fresh sensory note, and also distinguishing Iberian from non Iberian hams, the latter having higher concentrations. 

#### 2.2.5. Acids

Three main carboxylic acids were identified in the headspace of the samples. The acids, previously described by other authors [36,35], are probably products of the oxidation of aldehydes, though they may also be originated from enzymatic lipolysis during ham ripening [38]. The concentrations of acids were quite similar in the four locations with the exception of a higher concentration of hexanoic acid in SF. Those concentrations were lower than the OT for many of the non Iberian hams, unlike for Iberian hams, probably because the latter were subjected to long ripening.

#### 2.2.6. Furans

Furans have a significant impact on the aroma of dry-cured hams [24] because of their low OT and their pleasant aroma [30]. Thus, 2-pentylfuran has been found in all the locations, its concentration being slightly higher in SF probably because it derives from the autoxidation of linoleic acid [39]. It contributes to the aroma (green fruity and butter odor) of all the analyzed hams due to its low OT, and it also allows for distinguishing Iberian from non Iberian hams (Table 2). On the contrary, 2-ethylfuran showed minor interest in classifying hams or contributing to aroma. We have not found other furanes identified by some authors [40].

#### 2.2.7. Esters

Esters are described in dry-cured hams at the end of the maturation process and it seems that NaCl concentration affects the ester production through the activation of esterases [23]. Esters can be formed from the interaction of free fatty acids and alcohols by lipid oxidation in the intramuscular tissue so that the higher the content of alcohols, the higher the concentration of esters. We have quantified only an ester, butyl acetate, which contribution to ham odor is negligible due to its high OT.

#### 2.2.8. Sulfur Compounds

Sulfur compounds mainly originate from amino acids via Strecker degradation and have been associated to undesirable aromas for dry-cured hams. The presence of these kinds of compounds in outer muscles has been already reported [41]. Only a sulfur compound, dimethyl disulphide, has been quantified in very low concentrations in the four locations studied, and its presence could come from the degradation of sulfur amino acids due to a microbial deamination [42]. Fine correlations have been found between the concentration of dimethyl disulphide in the muscles BF and ST with taurine: 0.68 for ST and 0.59 for BF with p < 0.05. Those values increase up to 0.71 for ST, 0.79 for SF and 0.73 for BF, if the data are from Iberian hams only.

### 2.3. Effect of Breed on Dry Cured Ham Aroma

Once the differences in volatile composition between the locations of the ham slices were established, the next step in the study was to select those compounds that were able to distinguish samples by breed type (Iberian *vs.* non Iberian ham). Table 2 shows the mean concentrations of the volatile compounds quantified in Iberian and non-Iberian hams. These values corresponded to the average concentration for the studied muscles and subcutaneous fat. The Brown-Forsythe test [43] was applied to select those compounds that distinguished these two types of hams (*p* < 0.05). The selected volatile compounds were those with the codes 5–6, 13, 17–18, 21, 24–27, 29–31, 34, 36–38, 40–42 (Table 2). However, not all of them effectively contribute to dry cured ham aroma. In order to explain the differences in aroma of Iberian and non-Iberian dry cured hams, it is needed to take odor thresholds into consideration. Considering the odor activity value or OAV—as the ratio between the mean concentration of the volatiles and their odor thresholds, it was determined that 25 out of 46 volatiles (coded as 3, 6, 12, 15, 17, 21–28, 30–38, 40–42) could contribute to dry-cured aroma. Sixteen of these volatiles were aldehydes or alcohols. The highest concentrations of these compounds were mostly found in SF and SM muscle. The analyses by GC-O pointed out the association of fruity, woody, spicy and green fatty sensory descriptors with these volatiles in both kinds of hams, although their concentration was usually higher in Iberian hams. On the other hand, the relative importance of these volatile compounds also differed between the different locations. For example, in terms of OAV, 3-methyl-butanol is the major contributor to aroma in the three muscles, while in SF 2-heptanol had the highest OAV value. After 3-methyl-butanol, 2-heptanol was the second most relevant volatile compound according to their contribution to aroma in the three muscles, and in SF hexanal was the second most relevant volatile. 

## 3. Experimental

### 3.1. Samples

**Figure 2:**
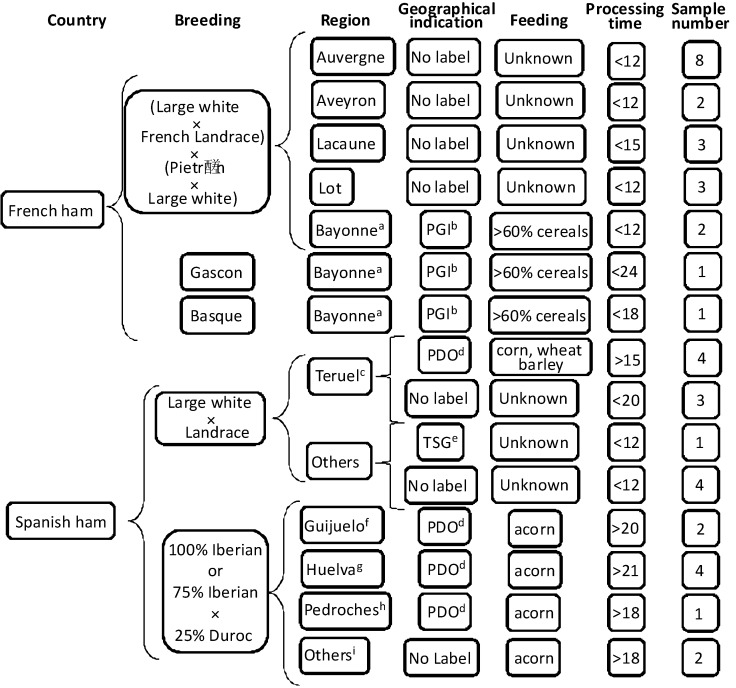
Information of the samples: country and region, geographical indication, basic breeding and feeding, approximately process time, number of samples, and sample codes.

*Notes*: ^a^ [44]; ^b^ PGI, Protected Geographical Indication; ^c^ Landrace, Large White, or (Landrace × Large White) × Duroc [45]; ^d^ PDO, Protected Designation of Origin; ^e^ TSG, Traditionally Speciality Guaranteed, Serrano [46]; ^f^ 100% Iberian breed or 75% Iberian × 25% Duroc-Jersey [47]; ^g^ 100% Iberian breed or 75% Iberian × 25% Duroc-Jersey [48]; ^h^ 100% Iberian breed or 75% Iberian × 25% Duroc and Duroc-Jersey [49]; ^i^ BOE, 2012 [50].

The samples in this study were forty-one dry cured hams from Iberian breeds and other commercial lines and crosses, including hams from France and Spain. Figure 2 shows together with the codes associated to each sample, information about their the pig breeds, together with their geographical origin and indication, feeding, maturation time, *etc*. Thirty-one hams were from non-Iberian pigs of several crossbreeds—(French Landrace × Large White) × (Piétrain × Large White), (Duroc or Landrace) × (Landrace or Large White or Landrace × Large White) and Landrace × Large White crossbreed sows mated with several genetic types, eight hams were from Iberian pigs—100% Iberian pig or Iberian × Duroc—Jersey with a minimum of 75% Iberian pig , and two hams were from Gascon and Basque pigs although crossed with Large White and other genetic types. The hams were processed by local manufacturers using the traditional method of each geographical origin, some of them being described in regulations [44,45,46,47,48,49,50].

### 3.2. Analysis of Volatile Fraction by Gas-Chromatography

**Figure 3:**
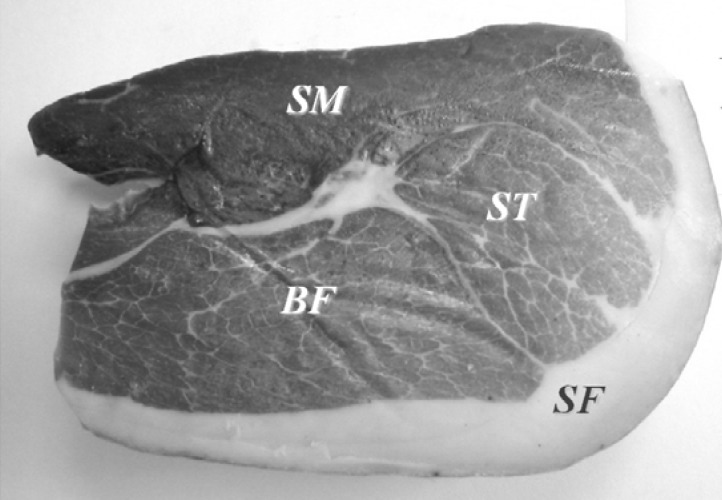
Locations of dry cured ham analyzed in this study: *Semimembranosus* (SM), *semitendinosus* (ST), and *biceps femoris muscles* (BF), and subcutaneous fat (SF).

A sample of approximately 350 g of the part located along and behind the femur was collected from each ham. The samples were stored in vacuum plastic bags at 5 °C until they were required for the analytical studies. A cylindrical stainless steel tool specially designed for ham sampling was used to extract approximately 5 g along the sample thickness. The samples were collected from 4 well differentiate locations: *biceps femoris* (BF), *semimembranosus* (SM) and *semitendinosus* (ST) muscles and subcutaneous fat (SF) (Figure 3).

Three grams of the minced hams were placed in 20 mL glass vials, tightly capped with a PTFE septum, and left for 10 min at 40 °C to allow equilibration of the volatiles in the headspace. The septum covering each vial was then pierced with a solid-phase micro-extraction (SPME) fiber coated with Carboxen/PDMS/DVB (Supelco, Bellefonte, PA, USA). This fiber was exposed to the headspace for 180 min. The temperature and time were automatically controlled in a Combipal (CTC Analytics AG, Zwingen, Switzerland) by the software Workstation v.5.5.2 (Varian, Walnut Creek, CA, USA).

When the adsorption process was completed, the fiber was inserted into the injector port of the GC for 5 min at 260 °C with the purge valve off (splitless mode). The compounds were separated in a DB-WAX column (J&W Scientific, Folsom, CA, USA; 60 m × 0.25 mm i.d. × 0.25 µm film thickness) installed on a Varian 3900 gas chromatograph (Varian) equipped with a flame ionization detector. The carrier gas was hydrogen. The oven temperature was held at 40 °C for 4 min and programmed to rise at 1 °C/min to a temperature of 91 °C, and then to rise at 10 °C/min to a final temperature of 201 °C, where it was held for 10 min to eliminate the memory effect. Each sample was analyzed in three replicates. The content of each volatile compound was calculated from the FID area and expressed as area units. A solution of 4-methyl-2-pentanol (1.2 mg/kg) was used as external standard in order to standardize the results of all the analyses. Thus, the quantitative result (mg/kg) of each volatile compound was computed by relating the peak area of the volatile compound to the area of external standard but taking into account the sample weight and the response factor of each volatile. 

The identification of volatile compounds by GC-MS (Table 2) was carried out on a 5975 Agilent Technologies Series MSD (Santa Clara, CA, USA) coupled to a gas chromatograph (7820A Agilent Technologies). Column and analytical conditions were identical to those described for gas chromatography, included the carrier gas. Volatile compounds were identified by the WILEY 7 library (John Wiley & Sons Limited, Hoboken, NJ, USA). The identified volatiles were purchased from Sigma-Aldrich (St. Louis, MO, USA), when possible, and the results validated with GC. These standards were used for determining their odor thresholds and their odor sensory descriptors in a matrix of fully deodorized olive oil. The standard solutions were also used for determining the response factors using the same deodorized olive oil. 

### 3.3. Response Factors

Concentrations in the range of 0.1–5.0 mg/kg and 0.5–20 mg/kg were analyzed under the analytical conditions described above. These two ranges allowed analyzing the recovery of volatile compounds at two different concentration levels. The absolute response factors of the standard compounds were calculated as the slopes of the linear regressions obtained from the ratio of total peak area as a function of concentration (averaged value from the two studied ranges). Relative response factors were obtained as the ratio of the absolute response factor of each compound to that of the internal standard (4-methyl-2-pentanol). 

### 3.4. Odor Threshold of Volatile Compounds

Fully deodorized edible oil was the matrix for the assessment of the odor threshold values; the absence of volatile compounds in the matrix was checked by the SPME-GC procedure described above. The sensory assessment was carried out in a test room arranged for evaluating sensory characteristics. Five assessors with a large experience of odor recognition in fat food products carried out the evaluation. Three samples were presented to the assessors following the triangle test whose results were statistically analyzed. Each sample (15 mL) was kept in standardized glasses at 29 °C ± 2 °C for 15 min and then tested. The samples were diluted until none of the assessors was able to classify the samples by odor intensity. The odor activity values (OAVs) of the volatile compounds, defined as the ratio of the concentration to the odor threshold [33], were calculated to determine their sensory significance. Thus, only those volatiles with OAV ≥ 1.0 contribute to the sensory perceptions [33].

### 3.5. GC-Olfactometry (GC-O)

GC-O was applied to assess the aroma notes corresponding to ham volatile compounds. It was performed with a GC (Varian 3900) connected to an olfactory port (OP275; GL Sciences Inc., Tokyo, Japan). Purified helium (purity > 99.99%) was used as the carrier gas at a constant flow rate of 3.8 mL/min. The inlet pressure was 170 kPa, and the inlet system was in split/splitless mode. The oven temperature program was that already described in a previous section. The effluent of the GC column was split 1–10 to the detector and the sniffing port, respectively.

The olfactory detection was performed during the chromatographic separation by three assessors with a large experience in odor recognition and sniffing of dry-cured hams. Damp air was continuously passed through the head of the port during operation; the flow rate was 30 mL/min. Elution of each aroma compound through the sniffing port was recorded by writing the beginning and end of the entire sensation of any odorant as well as its odor properties. The final aromagram (sensory description *vs.* Rt) is the result of merging the information from the individual analyses of the assessors. Table 2 shows the perception of the assessors (GC-O) at retention times. 

### 3.6. Amino Acids and Related Compounds

The analysis of amino acids and related compounds was carried out according to the method described by Ruiz *et al.*, 1999 [10]. Samples with an internal standard (norleucine 10 mg/mL) were mixed with 5% sulfosalicylic acid and homogenized with an Omnimixer. Homogenized samples were stored at 4 °C for 15 h and they were centrifuged at 15,300 g for 10 min and filtered through Whatman No. 54 paper. The pH of the filtrates was adjusted to 6 with 4 N NaOH, and then 50 μL were added to 200 μL solution of ethanol-water-triethylamine-phenyl tioisocyanate 7:1:2:1, and after 10 min the mixture was evaporated at cold temperature for 20 min, and reconstituted with a 0.5 M sodium phosphate buffer (500 μL), pH 7.4 and 5% acetonitrile. An aliquot (20 μL) was injected onto the HPLC system (LaChrom Elite, Tokyo, Japan), equipped with a UV diode array detector. The column was a Supelcosil LC-18 containing octadecyldimethylsilyl (25 × 4.6 mm; 5 μm particle size; Supelco), maintained at 35 °C. The gradient elution, at a flow rate of 1.0 mL/min, was achieved by using the following mobile phases: 0.03 M sodium acetate and 0.05% triethylamine, pH 6.80 (solvent A), and 90:10 acetonitrile-water (solvent B). The solvent gradient was programmed as follows: initial 96.8% (A)-3.2% (B) for 0.5 min; from 96.8 (A)-3.2% (B) to 95.5 (A)-4.5% (B) in 5 min; 90% (A)-10% (B) in 9.5 min; 81% (A)-19% (B) in 7 min; 73% (A)-27% (B) in 10 min; 1% (A)-99% (B) in 5 min.

The chromatographic signals were obtained at 254 nm. The identification and response factors were based on the analysis of solutions (1 mg/mL) of standard amino acids obtained from Sigma Chemical: L-Ala, L-Arg HCl, L-Asn, L-Asp, L-Cys, L-Glu, L-Gln, Gly, L-His HCl, Pro (4-OH), L-Ile, L-Leu, L-Lys HCl, L-Met, L-Phe, L-Pro, L-Ser, L-Thr, L-Trp, L-Tyr and L-Val. Creatine and creatinine were analyzed by using the method described by Mora *et al.* [51].

### 3.7. Statistical Analyses

Univariate and multivariate algorithms have been used by means of Statistica 8.0 (Statsoft, Tulsa, OK, USA). An ANOVA allowed selecting the volatile compounds and amino acids that better characterize the samples by breed. Correlation was used to determine the relationship between sensory attributes and chemical compounds while the first screening of the relationship between those two set of variables was carried out by principal component analysis (PCA).

## 4. Conclusions

The results comparing the volatile composition of Iberian and non Iberian hams showed that 20 volatile compounds had significant differences (*p* < 0.05) in their concentration depending on the combined effect of breed, feeding and curing process. Sixteen out of these 20 compounds have a sensory impact on dry cured ham aroma given that their concentrations were higher than their odor threshold. The amino acids composition of Iberian and non Iberian hams were also different for all the amino acids excepting tryptophan and asparagine. In this case, the concentration values were higher in Iberian hams in all cases probably due to their longer curing process compared to the other samples. Carnitine analyses showed, on the contrary, lower concentration values for Iberian hams due to the degradation of this compound during the processing [51]. The correlation between volatile compounds and amino acids provided satisfactory results (adjusted-R^2^ > 0.70) when only Iberian hams were including in the data set, the highest correlation coefficients corresponding to alcohols and aldehydes. The branched alcohols and aldehydes, typically produced from amino acid degradation [29], gives a chemical support to the relationship established between amino acids and volatiles. In general terms, the effect of amino acids on aroma seem to be more evident in Iberian hams, which has a longer maturation time, and thereby amino acid release is more remarkable. In those hams, the relation between volatile and non volatile compounds is proven, although it is still difficult to determine quantitatively the contribution of amino acids to aroma in addition to the effect of lipid oxidation. 
